# Structural Health Monitoring System Based on FBG Sensing Technique for Chinese Ancient Timber Buildings

**DOI:** 10.3390/s20010110

**Published:** 2019-12-23

**Authors:** Shao-Fei Jiang, Ze-Hui Qiao, Ni-Lei Li, Jian-Bin Luo, Sheng Shen, Ming-Hao Wu, Ying Zhang

**Affiliations:** 1 College of Civil Engineering, Fuzhou University, Fuzhou 350108, China; luoyubinghua@126.com (Z.-H.Q.); linilei@foxmail.com (N.-L.L.); luojianbin110@163.com (J.-B.L.); s_shen@fzu.edu.cn (S.S.); zy6163@163.com (Y.Z.); 2 Institute of Structural Detection, Fujian Academy of Building Research Co., Ltd. 6, Fuzhou 350002, China; clevermouse11@163.com

**Keywords:** FBG sensing technique, SHM system, Chinese ancient Chuan-dou-type timber buildings

## Abstract

Due to the long-term service, Chinese ancient timber buildings show varying degrees of wear. Thus, structural health monitoring (SHM) for these cultural and historical treasures is desperately needed to evaluate the service status. Although there are some FBG sensing-based SHM systems, they are not suitable for Chinese ancient timber buildings due to the differences in architectural types, structural loads, materials, and environment. Besides, a technical gap in Fiber Bragg grating (FBG) sensing-based column inclination monitoring exists. To overcome these weaknesses, this paper develops an FBG sensing-based structural health monitoring system for Chinese ancient Chuan-dou-type timber buildings that aims at monitoring structural deformation, i.e., beam deflection and column inclination, temperature, humidity, and fire around the building. An in-situ test and simulation analyses were conducted to verify the effectiveness of the developed SHM system. To validate the long-term-operation of the developed SHM system, monitoring data within 15 months were analyzed. The results show good agreement between the developed SHM system in this paper and other methods. In addition, the SHM system operated well in the first year after its deployment. This implies that the developed SHM system is applicable and effective in the health state monitoring of Chinese ancient Chuan-dou-type timber buildings, laying a foundation for damage prognosis of such types of timber buildings.

## 1. Introduction

In more than 5000 years of cultural inheritance, Chinese ancient timber buildings have become a symbol of China. However, ancient timber buildings show varying degrees of wear due to the effects of long-term load, weather-beaten erosion, typhoons, and earthquakes. To ensure that these historical treasures continue to be safely used and permanently inherited, damage diagnosis allows no delay. Thus, the health status of ancient timber buildings is in desperate need of monitoring.

As one of the most effective ways to improve structural safety and to extend service life, SHM systems have been widely applied to many infrastructures, such as bridges, high-rise buildings, and water conservation projects [1,2,3,4,5,6,7,8,9,10,11,12]. However, due to the long-time service and natural and man-made disasters, different types of ancient buildings show different types and extent of diseases. The principal purpose of SHM sensor system development is to obtain data that is directly related and sensitive to wear or damage. As a result, sensors should be deployed at the locations and parts of infrastructures that are sensitive to damage. Although SHM systems have been successfully used in some ancient buildings [13,14,15,16], these systems are not suitable for every Chinese ancient timber building due to the differences in architectural types, structural loads, materials, and environment.

Over the past two decades, SHM systems have made a lot of progress, especially with the advanced sensing technology and sensors [17,18]. However, due to the gap of technical levels, different SHM systems lead to varying precision and applicability. Considering the long-time damage evolution of ancient timber buildings, SHM systems should have enough stability for long-term and real-time monitoring.

Fiber Bragg grating (FBG) sensors have gradually been used in ancient timber buildings in recent years due to their durability, immunity to environmental factors, and ease of distributed monitoring. Inaudi [19] discussed the application of an optical fiber-based monitoring system for a cracked church vault and long-time monitoring for a harbor quay wall. Faiciai [20] proposed a quasi-distributed FBG-sensor monitoring system for in-situ measurement and continuous deformation monitoring in a painted timber panel. Wang [21] developed an SHM system and validated the feasibility by an experiment on a Tibetan heritage building. Marsili [22] raised a monitoring methodology for on-site behavior analysis of fiber-reinforced timber buildings. After analyzing the above references, we find that most of the existing FBG sensing-based SHM systems only focus on beam deformation monitoring. For ancient timber buildings, however, the column inclination is also an evaluation index for the structural service status. The SHM systems mentioned above lack column inclination monitoring, and especially lack an inclination monitoring method based on the FBG technique. Besides, Chinese ancient timber buildings typically have special architectural features, such as mortise-tenon joints, resulting in unique mechanical properties [23]. Thus, the common deformation transformation algorithm of an FBG sensing-based SHM system, which may cause large measurement errors, is inactive for Chinese ancient timber buildings.

To overcome the mentioned weaknesses, the authors developed a macroscopic deformation measurement technique for Chinese traditional timber buildings based on FBG sensors [23]. For the purpose of applying such a technique to actual engineering, this paper developed an SHM system based on the technique and applied it to the Seven Literary World, a typical two-story Chinese ancient Chuan-dou-type timber building in Fujian Province of China. Although the accuracy and the feasibility of the deformation measurement technique has been certified in laboratory tests [23], whether such a technique is applicative for practical engineering still needs to be verified due to the differences in construction, environment, and human factors between the practical engineering and laboratory tests. Thus, before the SHM monitoring system can be officially put into use, an in-situ test was carried out to verify the system precision and applicability. To guarantee the reliability of the assessment, in addition to FBG sensors, electrical sensors were also integrated into the SHM system. Two data acquisition device systems, which can respectively demodulate the FBG sensors and electric signals with high accuracy, were used in the SHM system. A self-devised visual system based on Browser/Server (B/S) mode, which enables integrated visualization of two kinds of monitoring sensors, was included in the SHM system. This work provides a new method for condition monitoring of Chinese ancient Chuan-dou-type timber buildings, laying a foundation for the damage prognosis of this type of building.

## 2. Structural Health Monitoring System

### 2.1. Introduction of Seven Literary World

**Figure 1:**
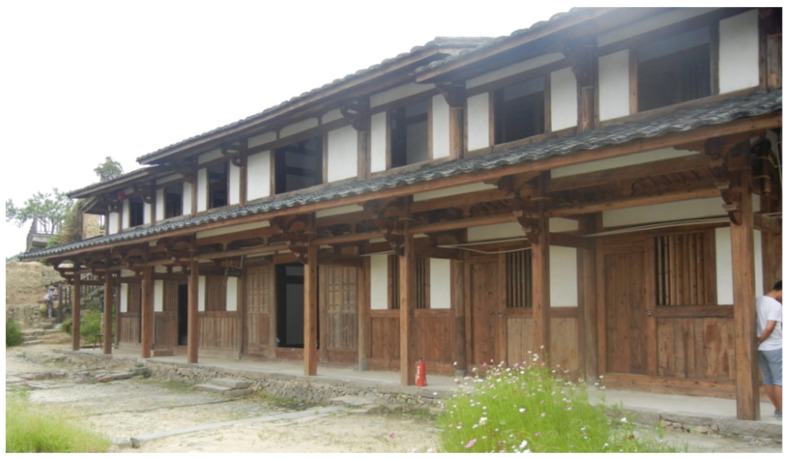
Seven Literary World.

**Figure 2:**
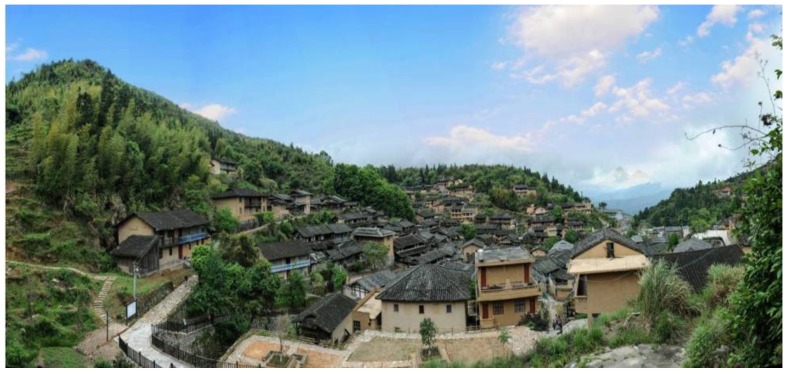
Guifeng Village.

**Figure 3:**
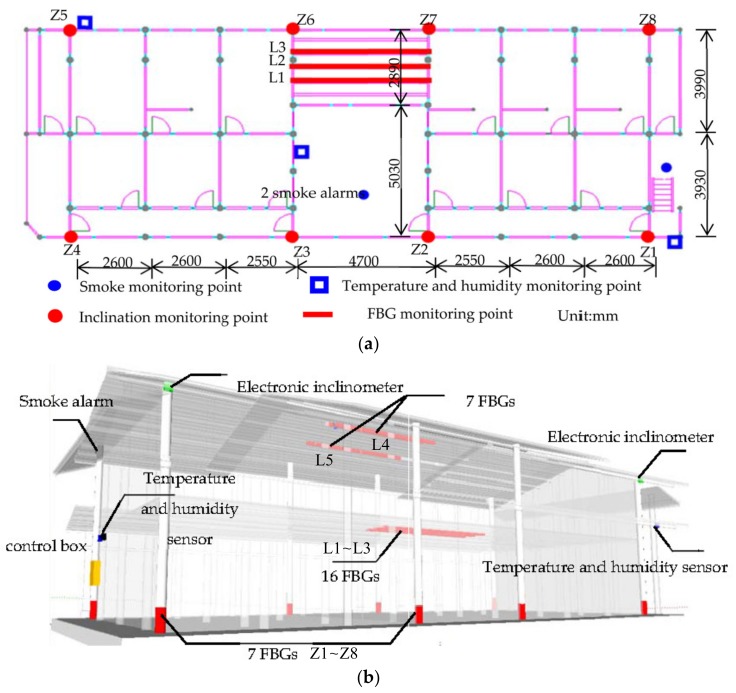
Layout of sensors in Seven Literary World: (**a**) Layout of sensors of the first floor plan; (**b**) Lateral view.

Seven Literary World, as shown in Figure 1, is located in the famous “historical and cultural village”, Guifeng village (shown in Figure 2), Fujian Province, China, which is a 257-year-old living fossil for studying the art of the Ming and Qing Dynasty. It is a two-story ancient timber dwelling with a rectangular and axisymmetric layout. The gross floor area of the building is 320 m^2^, and the detailed sizes of the building are shown in Table 1 and Figure 3. The structural bearing elements consist of columns, rafts, and grilles. The wall serves as a maintenance shield. As a typical Chuan-dou-type timber building, the columns and the architraves are connected by mortise-tenon joints.

However, owing to the influence of environmental erosion and human influence, this ancient timber dwelling has suffered from different types of damage, such as fungi corrosion and termite erosion. Local heritage buildings are in great need of protection. In 2012, a renovation for the ancient timber buildings was carried out under the local government organization, but the foundation soil was still unstable. Therefore, it is important role that an SHM system is developed to assess and predict those ancient settlements’ service states.

### 2.2. Composition and Deployment of the SHM System

Considering the factors of the large temperature difference between day and night as well as the high relative humidity caused by the geographical location, and the large structural deformation caused by great tourist flows, the SHM system deployed on Seven Literary World should include the following monitoring contents:

- Deflection of beam;
- Inclination angle of the column; and
- Temperature, humidity, and smoke around Seven Literary World.

Therefore, the entire system design included the following four components: Sensor subsystem, automatic collection and data transmission subsystem, data storage and management subsystem, comprehensive early warning, and structural safety assessment subsystem.

#### 2.2.1. Sensor Subsystem

The self-devised sensor subsystem of the SHM system consisted of 56 FBG sensors for monitoring the deflection of key beams and inclination angle of columns, 4 inclinometers for comparison with the columns’ inclination angle measured by the FBG sensors, 3 temperature and humidity sensors for monitoring the ambient temperature and humidity, and 3 smoke alarms for monitoring the smoke alert, as shown in Figure 3.

The span-to-height ratio of the 5 tested wooden beams in Seven Literary World is over 25. According to the authors’ research results [23], 5 FBG sensors were deployed on each beam at equal intervals. Correspondingly, 2 FBGs were installed in each position to function as a temperature compensation. As for the 8 tested columns, 3 FBG sensors were arranged on each wooden column. To avoid the most unfavorable conditions and to facilitate the installation and processing of subsequent data, FBG sensors were attached to the surface of the column at an equal angle of 60 degrees. Four electronic inclinometer sensors, which were used to verify the inclination from FBG sensors, were installed on the corner columns, Z1, Z4, Z5, and Z8, as shown in Figure 3a. The deployment of electrical sensors is depicted in Figure 3. The models and specifications of each sensor used in the sensor subsystem are shown in Table 2.

#### 2.2.2. Automatic Collection and Data Transmission Subsystem

**Figure 4:**
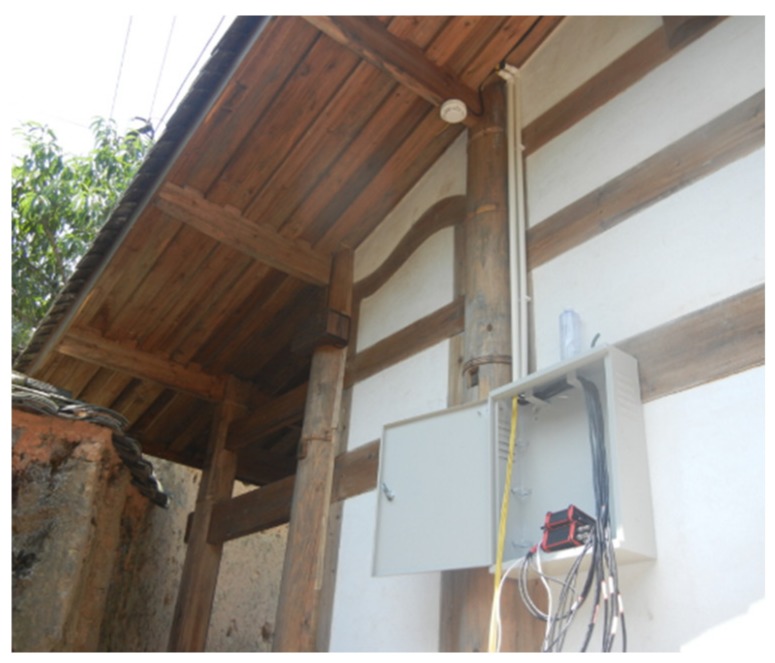
Terminal integrated system of the ancient timber building.

The functions of this subsystem are the acquisition of sensor data, transmission of data, and demodulation of signals. To simultaneously acquire, transmit, and demodulate all data, two sets of back-end integrated systems were arranged in the control box (i.e., terminal integrated system), as shown in Figure 4. One of them is a fiber-optic integrated system that integrates data from all FBG sensors and the other is an electric sensing system, responsible for integrating all data from electrical sensors, i.e., electronic inclinometer, temperature, and humidity sensor.

**Figure 5:**
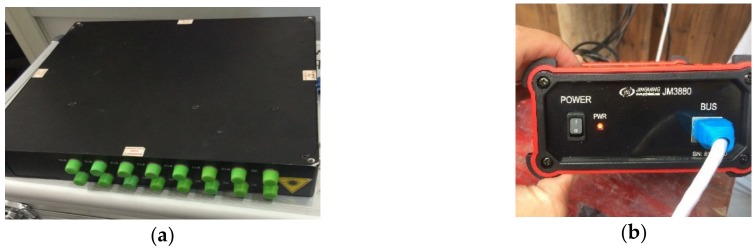
Signal demodulators: (**a**) Fiber grating demodulator; (**b**) Yangzhou Jingming Static Data Acquisition Instrument.

Through the fiber-optic integrated system, all FBG sensors’ signal are transmitted via Ethernet and demodulated by a fiber grating demodulator (shown in Figure 5a). Meanwhile, all electric signals are transmitted through the SIM card with a mobile network and are demodulated by Yangzhou Jingming Static Data Acquisition Instrument (shown in Figure 5b).

#### 2.2.3. Data Storage and Management Subsystem

**Figure 6:**
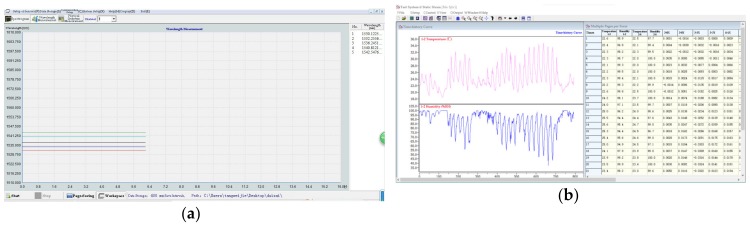
Data processing and control software: (**a**) Optical Sensor Analysis (OSA) software; (**b**) Yangzhou Jingming data acquisition software.

**Figure 7:**
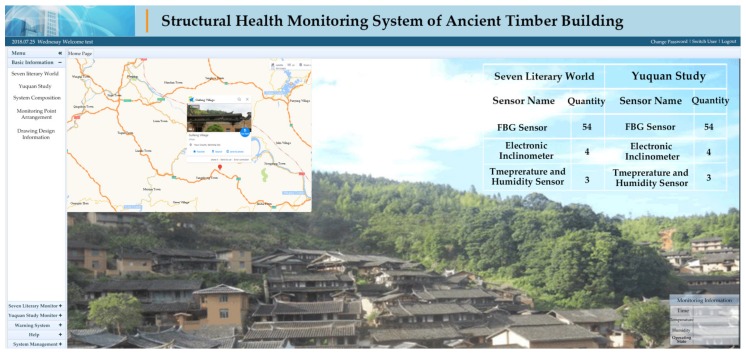
Structural Safety Assessment System server of the monitoring of an ancient timber building.

The data storage and management subsystem plays an important role in the entire system. The reason for it is that the subsystem possesses three functions, namely completing the verification of the monitoring, controlling the abovementioned monitoring and collecting process, and structured storing and managing of the obtained data. The data management included collecting, processing, analyzing, displaying, archiving, and storing all signals. Those works were mainly performed by two kinds of data processing and control software installed on the computer. The two types of software were Optical Sensor Analysis (OSA) software and Yangzhou Jingming data acquisition software as shown in Figure 6, respectively. The former manages the data from FBG and the latter manages the electric signals. After being processed and analyzed, the data is sent to the Structural Safety Assessment System server (shown in Figure 7) for structural safety assessment and generation of monitoring/assessment reports.

#### 2.2.4. Comprehensive Early Warning and Structural Safety Assessment Subsystem

**Figure 8:**
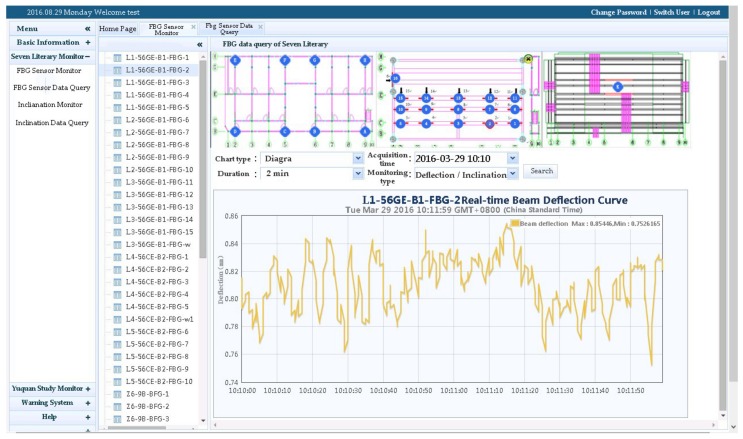
Real-time field monitoring of the ancient buildings via Structural Safety Assessment System server.

As shown in Figure 8, the subsystem can remotely monitor and analyze the required field signals, i.e., the deflection of the beam, inclination of the column, temperature, humidity as well as the smoke in the environment where the building is located. To achieve this goal, the subsystem must fully and reasonably coordinate various parts of the hardware and software systems, make full use of the building health state database based on the above monitoring information, and consider the changes of the environment and accidental load conditions. In other words, the subsystem enables real-time monitoring, which includes structural geometric deformation monitoring, structural static and dynamic responses monitoring, and structural member bearing capacity change monitoring, and signal visualization. Therefore, the operational state of the building can be assessed, and decisions based on the grading and warning of the structural status can be made.

## 3. Validation: In-Situ Test

The deformation algorithm employed in the FBG-based SHM system developed in this paper has been verified in laboratory tests [23]. Nevertheless, due to the large differences in construction, environment, and human factors between the practical engineering and laboratory tests, it is necessary to verify whether such an algorithm is applicable to practical engineering. Besides, the effectiveness of the SHM system for practical engineering also needs to be verified to ensure the credibility of the deformation data collected from the SHM system.

### 3.1. Deformation Monitoring

Two in-situ tests were carried out to verify the effectiveness of the SHM system: (1) For beam deflection, the effectiveness was verified by an in-situ sandbag loading test; and (2) for column inclination, the effectiveness was verified by the data comparison between the long-term monitoring and electronic inclinometer.

#### 3.1.1. Beam Deflection Monitoring

**Figure 9:**
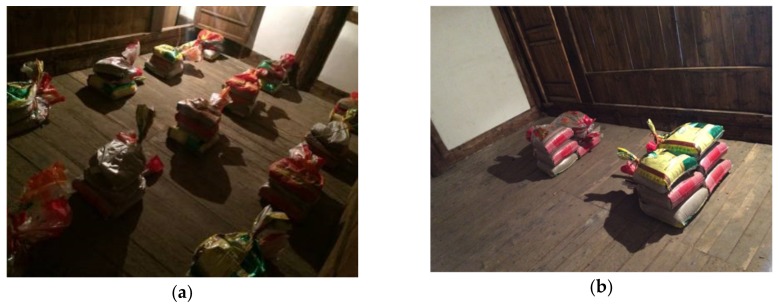
In-situ loading test: (**a**) Uniform loading; (**b**) Concentrated loading.

Taking three beams at the opisthodome of the first layer as the research object, the in-situ loading test was carried out. Considering that there was a lot of fine sand around the site, sandbags filled with sand were employed to provide loads. The loading methods were divided into two modes: Uniform loading and trisection-point concentrated loading, as shown in Figure 9. Five levels were divided for each loading mode, as shown in Table 3. To avoid tourists’ interference, the in-situ test was carried out at night. The sandbag banking process should be as gentle as possible to reduce the interference of floor vibration to the readings of LVDTS. To ensure the accuracy and stability of data, the data collection of each loading level was conducted 30 s after loading.

**Figure 10:**
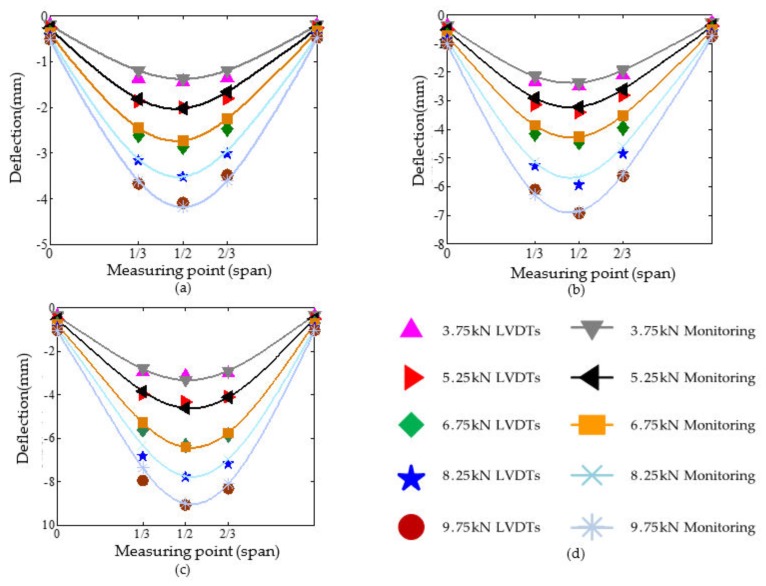
The comparison of beam deflection under uniform loading: (**a**–**c**) Comparison of L1~L3, respectively; (**d**) Legend of L1~L3.

**Figure 11:**
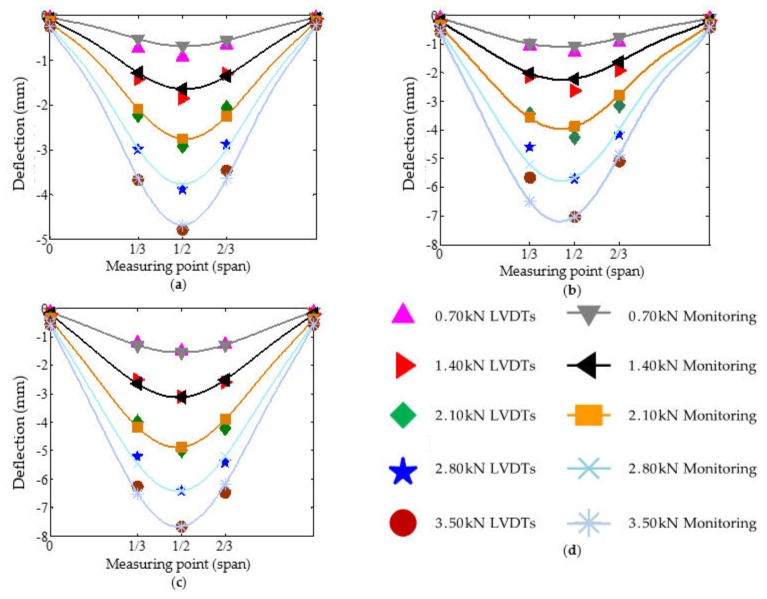
The comparison of beam deflection under concentrated loading: (**a**–**c**) Comparison of L1~L3, respectively; (**d**) Legend of L1~L3.

To verify the accuracy of the monitoring data, three electronic linear variable differential transformers (LVDTs) were installed on the bottom 1/3, 1/2, and 2/3 lengths of each beam during the in-situ test. The comparison between the static response data collected by the LVDTs deployed in the field and the measurement results from the FBGs of the SHM system is shown in Figure 10 and Figure 11.

In general, both the monitoring data and the LVDTs data have a longitudinal symmetric distribution. The maximum deflection appears in the midspan of the beam. Besides, the measured beam deflection from LVDTs is in good agreement with the monitoring deflection from FBGs. Although some errors exist, the maximum error does not exceed 10%. The reason for errors is that the Euler beam theory, the algorithm theory employed in the monitoring system, does not consider the influence of shear deformation on the beam deflection. The greater the span-depth ratio is, the smaller the effect of shear deformation on beam deflection. Thus, the accuracy of the algorithm increases with the increase of the span:depth ratio of beams. The maximum error appears at 8.25 kN under uniform loading and 3.50 kN under concentrated loading. This is because the vibration caused by wind during the measurement process interferes in the instrument measurement. In brief, the beams’ deflection deformation monitoring by FBGs is feasible and effective. The monitoring system can be applicable to different load levels and loading modes.

#### 3.1.2. Column Tilt Monitoring

**Figure 12:**
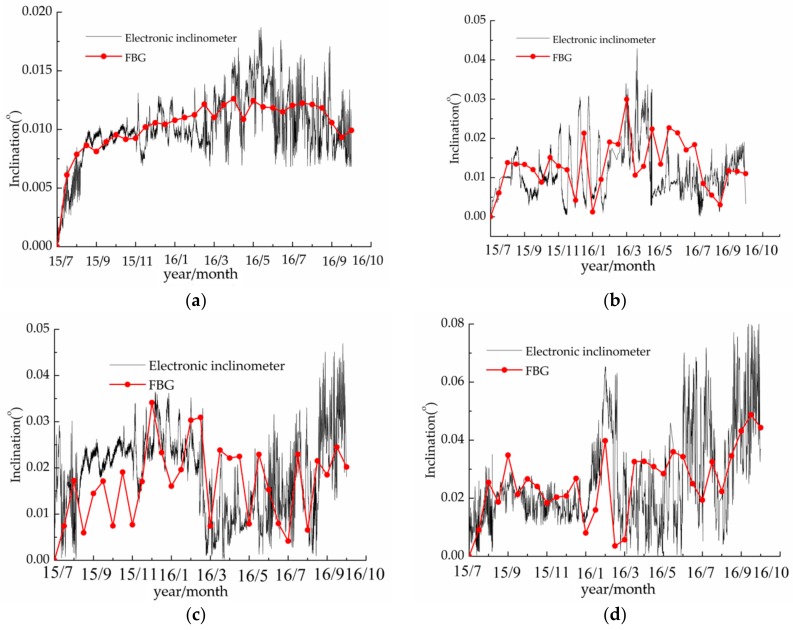
(**a**–**d**) respectively indicate column Z1, Z4, Z5, and Z8 inclination angle comparison curve.

The column inclination monitoring was validated by comparing the data collected by electronic inclinometers and the results from FBG sensors within 15 months. The deformation data were collected every 15 days. In order to minimize the environmental interference on data, the acquisition time was about 20:00. The comparison results are shown in Figure 12.

Comparing the curves obtained by connecting discrete data measured by the monitoring system every 15 days and the real-time data measured by electronic inclinometers, it can be seen that the overall trend of the inclination angle measured by the two methods is relatively close and both are in the same magnitude level. The difference between the two curves is that the data measured by electronic inclinometers show a certain fluctuation while the data monitoring by FBG sensors show relatively slight oscillation and most of them are within the data intervals measured by electronic inclinometers. This may be contributed to the difference of the sample frequency. It should be noted that only judging from Figure 12, there are some errors between the data measured by the two methods during the June 2015–November 2015. However, for the measurement of slight deformation (on the 0.01° magnitude level), the instrument and test operation need very high precision, which is difficult to achieve due to the slight vibrations caused by wind, human activities, etc. during monitoring. Besides, the inclination amplitudes of columns measured by the two methods are mostly between 0.01° and 0.05°. Therefore, it can be considered that the FBG sensing-based column inclination monitoring method is feasible and effective.

From the above two aspects, the deformation monitoring of the SHM system is feasible and effective.

### 3.2. Temperature and Humidity Monitoring

To verify the effectiveness of the temperature and humidity monitoring of the SHM system, a mechanical damage evolution model of the Seven Literary World was modelled through the general finite element (FEM) software Abaqus. The simulated results were compared with the survey results. The modeling process is divided into three steps: (1) Establish a user subroutine of local cyclic temperature and humidity conditions; (2) establish the heat and humidity conductive model; and (3) establish a mechanical damage evolution model.

#### 3.2.1. User Subroutine of Local Cyclic Temperature and Humidity Conditions

**Figure 13:**
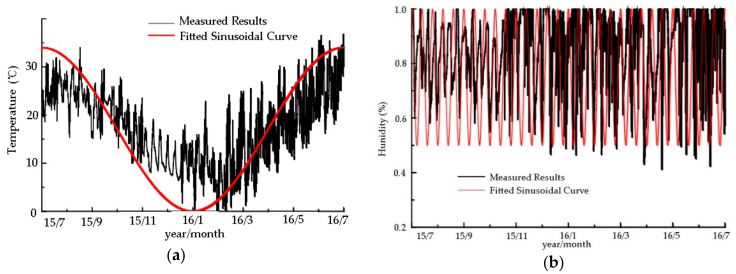
Fitted curves of temperature and humidity: (**a**) Temperature fitted curve; (**b**) Humidity fitted curve.

By analyzing data collected by temperature and humidity sensors (shown in Figure 13), we derived and fitted two sinusoidal functions for local temperature and humidity conditions:

(1) $$T\left(t\right)=17-17\sin\left(2\pi t/365-0.5\pi \right)$$

(2) $$u\left(t\right)=75+25\sin\left(2\pi t/12.17+0.5\pi \right)$$

where *t* is time, *T* is temperature, and *u* is humidity.

To simulate the external environment and predict the performance of the timber frame model, the two sinusoidal functions were programmed and embedded into the Abaqus FEM software as a user subroutine.

#### 3.2.2. Heat and Humidity Conductive Model

The scholar of the former Soviet Union Luikov [24] proposed a set of coupled differential equations in the 1960s to describe the phenomenon of heat and humidity transfer in porous materials. The Luikov model is applicative to almost all porous materials and comprehensively takes into account various factors, thus a simplified mathematical model applicable to timber was established based on the Luikov model [25]:

(3) $$\left\{\begin{array}{l} \rho C\frac{\partial T}{\partial t}=\nabla \cdot \left(\lambda \cdot \nabla T\right)\\ \frac{\partial u}{\partial t}=\nabla \cdot \left(D_{m}\cdot \nabla u\right) \end{array}\right.$$

where *ρ* is the oven-dry density of timber; *C* is the specific heat of timber; *T* is the temperature of timber (Unit: °C); *u* is the humidity content of timber; *λ* is the thermal conductivity of timber; *t* is time; and *D_m_* is the mass diffusion coefficient of timber, determined by:

(4) $$D_{m}=8.64\times 10^{-7}e^{4u}$$

Due to the conclusiveness of the boundary conditions on the flow and heat transfer characteristics as well as the necessity of the solution method for the mathematical model, appropriate boundary conditions were selected:

(5) $$\left\{\begin{array}{ll} T_{surf}=T_{a} & \mathrm{at}\Omega \\ \frac{q_{n}}{\rho }=S_{m}\left(u_{air}-u_{surf}\right) & \mathrm{at}\Omega \end{array}\right.$$

where *T_surf_* is the surface temperature of timber (unit: °C); *u_surf_* is the surface humidity content of timber; *T_a_* is the temperature of the ambient environment (Unit: °C); *q_n_* is the mass flow flowing through the timber surface; Ω is the boundary surface of the computational domain. *S_m_* is the humidity divergence coefficient; *u_air_* is the equilibrium humidity content of timber. The *S_m_* and *u_air_* can be determined by:

(6) $$S_{m}=3.2\times 10^{-8}e^{4u}$$

(7) $$u_{air}=0.01\times {\left[\frac{-T’\cdot \ln\left(1-h\right)}{0.13{\left(1-T/647.1\right)}^{-6.46}}\right]}^{\frac{1}{110T^{-0.75}}}$$

where *T’* is the temperature of the environment (unit: K).

The simplified heat and humidity conductive model was programmed and embedded into the Abaqus FEM software as a user subroutine to simulate the temperature and humidity transfer.

#### 3.2.3. Mechanical Damage Evolution Model

**Figure 14:**
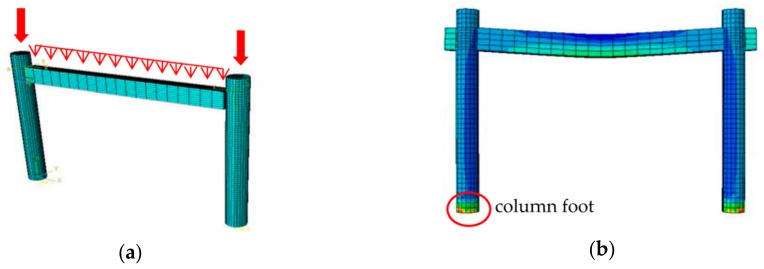
Lounge framework FEM (finite element method) model: (**a**) Loading model; (**b**) The von Mises stress contour plot.

On the basis of the research [26], a lounge timber frame model of the Seven Literary World was modeled through the FEM software Abaqus to analyze the damage evolution under the combined action of cyclic temperature and humidity conditions (shown in Figure 14a). The friction coefficient of the mortise-tenon joints of the model was corrected according to the real-time response measured by the monitoring system. To ensure the correctness of the model, the material parameters of the old timber, which was replaced during repair and subjected to material property testing, were adopted as the material properties of the model. The modeling process was divided into three steps:

(1) Establish the computation model of the heat and humidity transfer of the lounge timber frame, and calculate the time-history curves of the temperature and humidity distribution of the lounge timber frame. In the finite element software, the heat transfer analysis step was employed to simulate the transfer of temperature and humidity of the timber frame model. The outward surface temperature of the model was set to be consistent with the ambient temperature, and the mass flow flowing through the external surface of the model was set equal to the mass flow diverging to the timber surface due to the gradient difference between the humidity content in the external environment and the equilibrium humidity content in the timber. According to the abovementioned sinusoidal functions for local temperature and humidity conditions, the local maximum humidity reaches 100%, and the maximum temperature reaches 34 °C, so the initial humidity and temperature values of the model’s ambient environment were set to 100% and 34 °C.

(2) Establish the mechanical performance computation model of the lounge timber frame. The static general analysis step was used to calculate the mechanical properties of the timber frame model. Loads were applied by means of concentrated loading at the top of the column and uniform distribution loading at the beam.

(3) Import the time-history curve calculated in the step one into the mechanical performance computation model, and then apply external loads for computation.

Following the above three steps, the mechanical damage evolution model of the lounge timber frame was established, and the distribution of deformation and internal force were obtained under the combined action of loads and cyclic temperature and humidity conditions.

#### 3.2.4. Validation of Temperature and Humidity Monitoring

**Figure 15:**
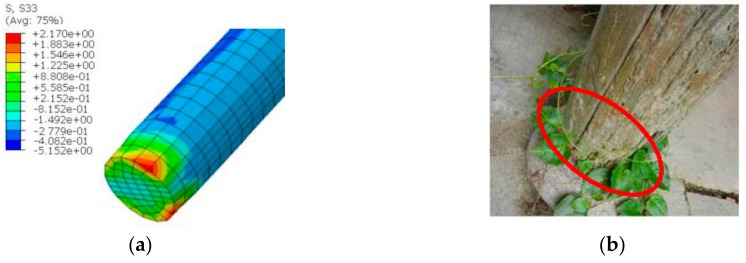
A column foot of the lounge: (**a**) Transverse grain stress contour plot of the column foot; (**b**) Site corroded column foot.

The transverse direction plastic stress contour plot of the column foot after 20 years of evolution was extracted as shown in Figure 15a.

The transverse grain stress S33 at the column foot’s edge fiber reaches 2.17 MPa, which is greater than 2.06 MPa in Table 1, Table 2 and Table 3 of [26]. It shows that the horizontal timber fibers at the edge of the lounge column foot are prone to cracking after 20 years of service, which is consistent with the result of the site survey (shown in Figure 15b). Therefore, it can be considered that the temperature and humidity monitoring of the SHM system is feasible and effective.

### 3.3. Smoke Monitoring

There is no doubt that smoke alarms can only be sold if they pass the product inspection. In addition, according to the fact that the building did not suffer from fire and that the smoke alarm did not warn, the smoke monitoring of the SHM system is feasible and effective.

## 4. Long-Term Monitoring Data Analysis

The sensor deployment time for deformation monitoring project was 1 July 2015. Taking the data on the completion date as the initial value of deformation, the deflection of five beams and the inclination of eight columns were measured. The monitoring period was from 1 July 2015 to 1 October 2016. The data was collected by the fiber-optic grating demodulator every 15 days, for a total of 31 times.

### 4.1. Beam Deflection

**Figure 16:**
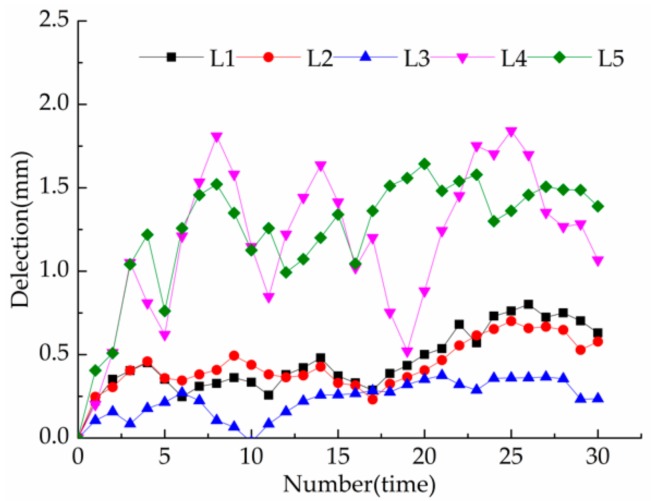
Beam deflection measurement curve.

Figure 16 shows the results of beam deflection monitoring. Although the deflection values show a fluctuating variation, due to the creep accumulation caused by long-term loads and humidity changes, the overall tendency increases slightly. The beam deflection increment is in the order of 1 mm, consistent with the status of no mutations and abnormalities during this time. Each beam holds the same length, but the deflection of L4–L5 is significantly larger than that of L1–L3. The main reason is that L4 and L5 serve as roof beams under a relatively higher load level. Thus, the L4–L5 are more vulnerable to damage during service, and must be monitored emphatically.

### 4.2. Column Titling

**Figure 17:**
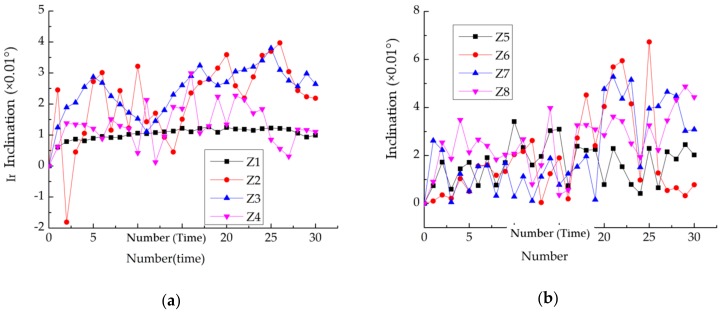
Column inclination measurement curve: (**a**) Z1–Z4 inclination measurement curve; (**b**) Z5–Z8 inclination measurement curve.

Figure 17 shows the results of column inclination monitoring. The inclination values show a fluctuating variation, but basically show an increasing tendency. The inclination increment is in the order of 0.01°, consistent with the current stable service state. Besides, the inclination direction of each column is substantially parallel to the weak axis. Thus, depending on the consistency of the inclination direction, the average value of column inclination can be used as an important indicator of the overall structural tilt.

Overall, the SHM system deployed at Seven Literary World operated well in the first year after its deployment. This SHM system can provide accurate data only by deploying sensors at five positions on the bottom of each beam and three positions at each column base, retrenching monitoring costs. Besides, this monitoring technology fills the technical gap in FGB sensing-based column inclination monitoring. This SHM system provides real-time and accurate monitoring data for its evaluation and maintenance during service, laying a foundation for the prognosis of Chinese ancient Chuan-dou-type timber buildings.

## 5. Conclusions and Remarks

This paper developed an SHM system for monitoring the in-service status of Chinese ancient Chuan-dou-type timber buildings based on the authors’ developed deformation monitoring methods [24]. Meanwhile, the SHM system also involves monitoring temperature, humidity, and fire disaster around the environment. A series of experiments were conducted to verify the applicability and effectiveness of the developed SHM system. The effectiveness of the system operation was verified by analyzing the long-term monitoring data. The concluding remarks are drawn as follows:

(1) This paper developed an SHM system that monitors the deflection of the beam, inclination angle of the column, temperature, humidity, and fire disaster around Seven Literary World. The system consists of four subsystems, i.e., sensor subsystem, automatic collection and data transmission subsystem, data storage and management subsystem, and comprehensive early warning and structural safety assessment subsystem.
(2) The accuracy of the employed algorithm and the applicability of the SHM system were verified through an in-situ test. The results indicated that the SHM system shows good precision and can accurately monitor the beam/column deformation of the Chuan-dou-type timber building.
(3) The long term-operation effectiveness of the SHM system was verified by analyzing the data monitored within 15 months, which indicates that the SHM system operated well in the first year after its deployment, laying a foundation for damage prognosis of Chuan-dou-type timber buildings.

The effectiveness and operation of the developed SHM system in this paper were validated by a limited number of experimental and finite element analysis results. More efforts need to be conducted in the future to make the SHM system more intelligent, convenient, precise, and efficient. It is believed that more and more SHM systems will be developed and applied to practical engineering, with the growing requirement in the field of structural health monitoring.

## Figures and Tables

**Table 1:** The plane size of Seven Literary World.

Name	Facial Width ^5^/mm	Throat ^6^/mm				
		First Floor (3.00 m High)		Second Floor (2.88 m High)		
		First Room	Final Room	Corridor	First Room	Final Room
Mingjian ^1^	4700	5030	2890	1100	3930	2890
Cijian ^2^	2550	3930	3990	1100	2830	3990
Shaojian ^3^	2600	3930	3990	1100	2830	3990
Mojian ^4^	2600	3930	3990	1100	2830	3990
Total length	20,200	7920				

^1~4^ The rooms from the middle to the outermost along the longitudinal axis of the building. ^5~6^ The room’s size along the longitudinal and horizontal axis of the building, respectively.

**Table 2:** The sensor models of the sensor subsystem.

Number	Sensor	Model	Quantity	Description
1	FBG of 300 mm gauge length	TXD-YF-030	56	Wavelength range (1510 nm~1590 nm); Precision (±2~3 με)
2	Electronic inclinometer	LE-60	4	Measuring range (0~90°); Bidirectional measurement
3	Temperature and humidity sensor	KSW-60	3	Measuring range (0~100%RH/−40~+60 °C); Precision (±2%RH/±0.3 °C)
4	Smoke alarm	/	3	/

**Table 3:** Load level in two loading cases (Unit: kN).

Cases	First Level	Second Level	Third Level	Fourth Level	Fifth Level
**Uniformly distribution load**	3.75	5.25	6.75	8.25	9.75
**Concentrated load**	0.70	1.40	2.10	2.80	3.50
